# Tear Lactoferrin and Lysozyme as Clinically Relevant Biomarkers of Mucosal Immune Competence

**DOI:** 10.3389/fimmu.2019.01178

**Published:** 2019-05-31

**Authors:** Helen G. Hanstock, Jason P. Edwards, Neil P. Walsh

**Affiliations:** ^1^Extremes Research Group, School of Sport, Health and Exercise Sciences, College of Health and Human Sciences, Bangor University, Bangor, United Kingdom; ^2^Swedish Winter Sports Research Centre, Department of Health Sciences, Mid Sweden University, Östersund, Sweden

**Keywords:** antimicrobial proteins (AMPs), common cold, contact lenses, dehydration, endurance exercise, infection risk, upper respiratory tract infection (URTI)

## Abstract

Tears have attracted interest as a minimally-invasive biological fluid from which to assess biomarkers. Lactoferrin (Lf) and lysozyme (Lys) are abundant in the tear fluid and have antimicrobial properties. Since the eye is a portal for infection transmission, assessment of immune status at the ocular surface may be clinically relevant. Therefore, the aim of this series of studies was to investigate the tear fluid antimicrobial proteins (AMPs) Lf and Lys as biomarkers of mucosal immune status. To be considered biomarkers of interest, we would expect tear AMPs to respond to stressors known to perturb immunity but be robust to confounding variables, and to be lower in participants with heightened risk or incidence of illness. We investigated the relationship between tear AMPs and upper respiratory tract infection (URTI; study 1) as well as the response of tear AMPs to prolonged treadmill exercise (study 2) and dehydration (study 3). Study 1 was a prospective cohort study conducted during the common cold season whereas studies 2 and 3 used repeated-measures crossover designs. In study 1, tear Lys concentration (C) as well as tear AMP secretion rates (SRs) were lower in individuals who reported pathogen-confirmed URTI (*n* = 9) throughout the observation period than in healthy, pathogen-free controls (*n* = 17; Lys-C, *P* = 0.002, *d* = 0.85; Lys-SR, *P* < 0.001, *d* = 1.00; Lf-SR, *P* = 0.018, *d* = 0.66). Tear AMP secretion rates were also lower in contact lens wearers. In study 2, tear AMP SRs were 42–49% lower at 30 min−1 h post-exercise vs. pre-exercise (*P* < 0.001, *d* = 0.80–0.93). Finally, in study 3, tear AMPs were not influenced by dehydration, although tear AMP concentrations (but not secretion rates) displayed diurnal variation. We conclude that Lf and Lys have potential as biomarkers of mucosal immune competence; in particular, whether these markers are lower in infection-prone individuals warrants further investigation.

## Introduction

Tears are an attractive medium from which to assess biomarkers. For several decades, tear biomarkers have been used as a tool to understand the mechanisms and consequences of ocular disease. However, in recent years, interest in the tear fluid has increased as a source of biomarkers for the detection of systemic disease and to assess neuroendocrine responses to stress (1–3). Evaluation of systemic immune status using the tear fluid may be relevant because transmission of viral upper respiratory tract infections (URTI) can occur at the ocular surface (4, 5), and transmission of pathogens via self-inoculation at the eyes or nose may even occur more readily than oral transmission (6). These observations suggest that it is important to maintain an effective immune defense at the eye to prevent both ocular and systemic infections, and also highlight the ocular surface as a site of clinical relevance for immune-monitoring applications.

Lactoferrin (Lf) and lysozyme (Lys) are two of the most abundant antimicrobial proteins (AMPs) in tear fluid (7) and play important roles in innate mucosal defense. Lf is an iron-binding glycoprotein that exhibits a multitude of antimicrobial activities, including antiviral activity against pathogens responsible for common URTIs such as respiratory syncytial virus (8) and influenza (9), as well as sequestration of iron to prevent bacterial growth and binding of Gram-positive and Gram-negative bacteria (10). Lys is a bacteriolytic enzyme discovered by Fleming (11) that exhibits antimicrobial activity predominantly by attacking the bacterial cell wall. Synergistic effects of Lys and Lf in neutralizing bacterial pathogens have also been reported (12). Besides the direct actions of AMPs against microorganisms, Lf and Lys may also play a role in modulating host immune responses to infections, for example by exerting anti-inflammatory effects at mucosal surfaces (13, 14). It is notable that the specific targets of Lys are typically bacteria, yet URTIs are most typically of viral origin (15). However, the broader role of Lys in immune modulation provides a rationale for its involvement in defense against a wider spectrum of pathogens.

For almost three decades, salivary secretory IgA (s-IgA) has been a favored, convenient biomarker of immune status, although an abundance of both positive and negative results as well as high biological variability has led its validity to be questioned (16). Salivary AMPs have also been identified as biomarkers of interest for the assessment of innate immune status and susceptibility to URTI (17). One study reported lower salivary Lys and Lf concentrations in elite rowers during 5 months' heavy training, compared to sedentary individuals (18), but to date no relationship between salivary Lf or Lys and susceptibility to URTI has been reported. Salivary Lys has, however, been linked to bacterial respiratory infection incidence in a clinical population; in a cohort of patients with chronic obstructive pulmonary disease, Taylor et al. (19) reported lower salivary lysozyme concentrations in infection-prone patients compared to those who reported fewer episodes of infection. Salivary Lys may also be associated with occupational stress (20), while salivary Lf is responsive to a passive laboratory stressor in tandem with increased vagal tone and modest increases in sympathetic activity (21). Taken together, these observations suggest that, at least in saliva, Lf and Lys secretion is sensitive to changes in autonomic activity as well as stressors such as physical activity, and may also have a prophylactic effect in the prevention of infection. Regardless, issues with saliva sampling remain, not least because saliva is the pooled product of secretions from multiple glands, each with its own autonomic innervation and regulation, where the secretions from the major glands differ substantially in their composition of proteins (22, 23).

The tear film is comprised of three layers, an inner mucin layer, middle aqueous layer and outer lipid layer. The outer lipid layer is secreted by the Meibomian glands and serves to prevent rapid evaporation of the aqueous layer. The aqueous component is predominantly secreted by the main and accessory lacrimal glands with minor contributions from corneal and conjunctival cells. Conjunctival goblet cells are the major contributor to the mucin layer. Lf and Lys are found in the middle aqueous component and are constitutively synthesized and secreted by lacrimal gland acinar cells at concentrations around 1–3 mg·ml^−1^ (7, 24), each comprising around 20–30% of total protein in basal and reflex tears (25). Basal tear flow rate has been reported at around 1 μL·min^−1^ but can increase more than 50-fold upon stimulation (7, 26). Lacrimal gland regulation occurs in three broad steps: activation of afferent nerves in the cornea and conjunctiva leads to stimulation of efferent autonomic nerves, which in turn activates cellular signaling pathways in the acinar and duct cells that lead to secretion of proteins, electrolytes and water (27). Tears have therefore been described as the final output of the lacrimal functional unit, integrating the major glands and interconnecting innervation (28). Lacrimal gland secretory activity is also modulated by steroid hormones, oxidative stress and inflammation (29–31). Pro-inflammatory cytokines; in particular IL-1β and TNF-*A*; can inhibit neurally-mediated lacrimation and have been implicated in several diseases characterized by disordered lacrimal gland secretory activity, such as dry eye disease and Sjögren's syndrome (31, 32). As well as providing a mechanism by which disordered lacrimation can arise, these observations suggest that lacrimal gland secretory activity may be sensitive to acute stressors (such as prolonged exercise or psychological stress) that exhibit brief, systemic immunomodulatory effects. This may enable the integrity of antimicrobial defense at the ocular surface to be used as a non-invasive indicator of the influence of daily life stressors on global immune status.

Considering that tear sampling is minimally-invasive in nature, and that advances in nanotechnology may permit development of wearable sensors in contact lenses (33), we suggest that there is potential for tear biomarkers to supersede salivary markers for monitoring applications in athletes, military personnel or other field-based scenarios where blood sampling is impractical. Indeed, we recently demonstrated that s-IgA in tear fluid is reduced prior to episodes of URTI (34) and is also temporarily depressed by prolonged, moderate-intensity exercise (34) and brief psychological stress (35). However, the potential of other tear biomarkers as diagnostic or monitoring tools for immune- or health-status remains relatively unexplored. Given that Lf and Lys are abundant in tears and that the eye is a clinically relevant site at which to evaluate host defense, investigation of the relationship between tear AMPs and URTI susceptibility is warranted.

The purpose of this series of studies was therefore to investigate the utility of tear AMPs to evaluate mucosal immune competence and URTI susceptibility in otherwise healthy, active individuals. First, to investigate whether tear AMPs could provide a clinically relevant measure of immune status, in a prospective cohort study we set out to investigate whether any differences in tear AMPs could be observed in participants who reported URTI vs. those who did not, and also whether any perturbations in tear AMPs arose in the days prior to URTI. Next, we investigated tear AMP responses to prolonged exercise, known to perturb mucosal immunity (36, 37), and dehydration, which has been commonly cited as a confounding variable that may decrease flow rates of mucosal fluids but does not affect systemic immunity (34, 38). To our knowledge tear AMP responses to these physiological stressors have not been previously investigated, thus, the purpose of studies 2 and 3 was to investigate tear AMP responses to prolonged exercise and dehydration. We postulated that if tear AMPs respond to each stressor as expected, this will not only indicate a direct effect (or no effect) of the stressor on key components of mucosal immune defense, but also improve confidence in tear AMPs as minimally-invasive biomarkers of systemic immunity. Collectively, these studies aimed to evaluate the utility of tear Lf and Lys as novel biomarkers of URTI susceptibility.

## Materials and Methods

### Overview

This investigation is an extension of a previous publication. Herein we present novel analyses of Lf and Lys from tear samples that have been previously been used to evaluate the utility of tear secretory IgA as a biomarker of URTI risk (34).

To evaluate the utility of Lf and Lys as biomarkers of immune status, we employed a multi-study approach. In study 1, we evaluated the utility of Lf and Lys to predict URTI in a cohort of recreationally active individuals during the common cold season. In study 2, we investigated the response of tear Lf and Lys to prolonged exercise. Finally, in study 3, we investigated the influence of hydration status on tear Lf and Lys. All studies received approval from the Bangor University School of Sport, Health and Exercise Sciences Ethics Committee (application numbers S/PhD08-14/15, S/PhD11-12/13 and S/PhD09-13/14). Participants provided written, informed consent before taking part, and all test procedures were conducted in accordance with the Declaration of Helsinki.

### Study 1

#### Participants

Forty participants (26 men and 14 women, age 22 ± 4 y, mean ± SD) were recruited to take part in the study during the fall common cold season. Participants were university staff and students, and the study took place within the first 6 weeks of the academic semester, a period where anecdotal reports suggest that URTI is highly prevalent in universities. Due to drop-outs and non-compliance, 33 participants completed the study. Participants reported that they had been free from upper respiratory symptoms (URS) for a month before participating, did not have any underlying health conditions (including no recent diagnosis or test for mononucleosis within 1 y), and were not taking medication known to influence immune indices. Eight participants wore contact lenses. Participants did not eat or drink (besides water) 1 h prior to tear sample collection.

#### Experimental Procedures

Detailed experimental procedures for this study have been published previously (34). Briefly, participants completed a 3-week URS-monitoring protocol. Participants provided a tear sample each week and reported URS daily online using the Jackson Common Cold Scale (39). The scale comprises a global question “Do you think you are suffering from a common cold today?” followed by eight symptom items scored either 0 (not at all), 1 (mild), 2 (moderate) or 3 (severe). If participants answered yes to the global question or reported a symptom score ≥ 6 for two consecutive days, they were deemed to have reported an episode of upper respiratory illness (URI) and were contacted by investigators and asked to report to the laboratory within 48 h. When participants with URI arrived at the laboratory we collected a tear sample as well as nasopharyngeal and throat swabs according to standard procedures (40). Swabs were used at a later date for laboratory detection of common viral and bacterial pathogens using real-time polymerase chain reaction methods as previously described (34). Participants that reported an episode of URI were asked to continue to report their symptoms daily until they had been free from URI for 4 weeks. Participants who did not report URI provided final tear samples as well as nasopharyngeal and throat swabs after 3 weeks of URS monitoring. To control for diurnal variation between sampling in the same individual, samples were collected after 10:00 to avoid rapid changes in tear composition that may occur in the period after waking. Participants provided subsequent samples as close to the original sampling time as possible and within a maximum 3 h window. All participants completed their 3-week URS monitoring period within a 6-week window in September-October.

### Study 2

#### Participants

Thirteen healthy, recreationally active male participants (age 23 ± 5 y, height 1.79 ± 0.08 m, BM 79 ± 9 kg, VO_2peak_ 52.8 ± 5.6 mL·kg^−1^·min^−1^) took part in the study. Participants were non-smokers, who had not taken prescription medication or used dietary supplements for 1 month before taking part in the study and did not wear contact lenses. For 24 h before each trial, participants were asked to refrain from caffeine, alcohol, over-the-counter medication and heavy exercise. Participants reported that they had been free from URS for 1 week before each trial.

#### Preliminary Tests

During the preliminary visit, participants completed a ramped treadmill exercise test to determine VO_2peak_ according to a protocol detailed in a previous study (36). Following the incremental test, a treadmill speed to elicit ~60% VO_2peak_ was interpolated from the integrated submaximal running stages in the ramped test using linear regression. After participants had rested for 30 min, this speed was then verified by measuring steady-state VO_2_ during the last minute of a 5 min exercise bout, with treadmill speed adjusted and the exercise bout repeated where necessary.

#### Experimental Procedures

Participants completed two experimental trials in a randomized, cross-over design, in accordance with a protocol described previously (34). For each trial, participants reported to the lab at 07:30 and were provided with a standardized breakfast and a fluid allowance of 35 mL·kg^−1^·day^−1^ *pro rata* for the pre-exercise period. During the exercise trial (EX), participants completed a 120 min treadmill run at ~65% VO_2peak_ in temperate conditions; a duration and intensity previously shown to perturb *in vivo* immunity (36). During the rested control trial, participants rested in an upright seated position for 120 min (REST). EX or REST commenced at 11:00. During EX only, participants reported Borg's rating of perceived exertion every 5 min and we collected 60 s expired gas samples at 10 min intervals for VO_2_ assessment. Expired gas was collected into Douglas bags and analyzed for O_2_, CO_2_ (Servomex 5200, Crowborough, UK), volume and temperature (Harvard Apparatus, Edenbridge, UK), enabling derivation of VO_2_ using the Douglas bag method. As the speed was fixed for the 120 min exercise period, actual exercise intensity during the 120 min rose from 62.7 ± 6.4% VO_2peak_ at 10 min to 67.9 ± 5.4% VO_2peak_ at 110 min due to VO_2_ drift. Heart rate (HR) was recorded at 5 min intervals throughout the 120 min exercise/rest period in both trials. HR and Borg data have been reported previously (34). During the 120 min run in EX, participants were provided with 3 mL·kg^−1^·h^−1^ plain water to offset fluid losses through sweating; during REST and non-exercising periods in EX, fluid intake was provided at a rate of 35 mL·kg^−1^·day^−1^. Tear samples were collected at five time points during EX: pre-exercise, post-exercise, 30 min post-exercise, 1 h post-exercise and 24 h post-exercise, and at the equivalent time points during REST.

### Study 3

#### Participants

Thirteen male participants (age 23 ± 4 y, height 1.81 ± 0.05 m, body mass (BM) 80 ± 10 kg, VO_2peak_ 56.4 ± 7.8 mL·kg^−1^·min^−1^) visited the laboratory on three occasions. Participants were non-smokers and none wore contact lenses. Participants avoided caffeine, alcohol, over-the-counter medication and strenuous exercise for 24 h before each trial. No episodes of upper respiratory symptoms (URS) were reported by participants during the week preceding each visit.

#### Preliminary Visit

On the first visit, participants completed a ramped maximal treadmill running test to determine their VO_2peak_, according to a test procedure previously described (34). The ramped test was followed by a 30-min treadmill walk at 4% gradient in an environmental chamber set to 40°C and 40% relative humidity (RH). The walking speed was interpolated from the ramped protocol and estimated to elicit 50% VO_2peak_ (mean: 6.6, range: 5.5–7.5 km·h^−1^). Nude body mass loss (BML) during the 30-min exercise bout was used to calculate a sweat rate for each participant (BML/exercise time in minutes). We then used the sweat rate to estimate the total walking duration required to elicit 3% BML for the main experimental trials.

#### Experimental Procedures

On the second and third visits, participants completed a dehydration (DEH) or euhydrated control (EUH) trial in a randomized, crossover design. Each trial lasted 27.5 h, from 08:00 on day 1 until 11:30 the following day. Standardized meals were provided at fixed time points throughout the trials. On the morning of day 1, participants were provided with fluids at a rate of 40 mL·kg^−1^·day^−1^ until 14:00. Beginning at 14:00 on day 1, participants performed three bouts of exercise in the chamber in the same environmental conditions as used in the preliminary test (40°C, 40% RH). The duration of each exercise bout was determined based on the sweat rate measured during the first test and estimated to elicit 1% body mass loss. Participants rested for 30 min outside the chamber (~ 18°C) in between exercise bouts. During DEH, participants received no fluids, whereas during EUH participants were provided with a volume of plain water equivalent to 1% BM during each bout. Core temperature was monitored continuously during the dehydration protocol using a rectal thermistor, to ensure participants' core temperatures did not exceed 39°C. After three exercise bouts, participants remained in the laboratory overnight. During this period, participants received a standardized evening meal at 18:30 and engaged in sedentary activities with restricted fluid intake (DEH: 4 mL·kg^−1^·day^−1^) or control fluid intake (EUH: 40 mL·kg^−1^·day^−1^) until they were permitted to sleep between 23:00 and 07:30. Tear samples as well as plasma samples for determination of osmolality were collected at four time points: 14:00 day 1 (0% BML), ~16:30 day 1 (~2% BML), 08:00 day 2 (~3% BML) and 11:30 day 2 (~0% BML). A standardized breakfast was given at 08:30 on day 2, coinciding with commencement of the rehydration period. BML and plasma osmolality data have been presented elsewhere (34).

### Tear Sample Collection, Handling and Analysis

Tear samples of at least 0.5 μL volume were collected from the inferior marginal tear strip into 10 μL glass microcapillary pipets using a technique previously described (34). Collection time was recorded by a second operator and the tear flow rate calculated as collection volume divided by collection time, assuming the density of tear samples to be 1.00 g·mL^−1^. Samples were then diluted 100x in phosphate-buffered saline and stored at −80°C until analysis. Commercially available ELISA kits (AssayPro, St. Charles, MO, USA) were used to determine concentrations of Lf and Lys in tear samples (mean intra-assay CVs: Lf 4.4%, Lys 4.8%).

### Statistical Analysis

Statistical analyses were performed using a combination of the Excel (Office Professional 2016, Microsoft, Redmond WA, USA), SPSS (v20, IBM, Chicago, IL, USA) and Prism (v7, GraphPad, San Diego, CA, USA) software packages. Data were checked for normality using the Shapiro-Wilk test and Q-Q normality plots. Where variables were log-normally distributed, statistical analyses were performed on data transformed as follows to avoid negative skew: x_transformed_ = log_10_(x+1).

In study 1, *t*-tests were used to determine group-wise effects for normally distributed variables; Welch's correction was applied in cases of unequal variance. One-way repeated measures ANOVA was used to compare within-subject effects. Results of studies 2 and 3 that employed repeated-measures crossover designs were analyzed using two-way repeated-measures ANOVA in SPSS. In cases where sphericity was violated, the Greenhouse-Geisser correction was applied and corrected degrees of freedom are displayed. Cohen's *d* effect sizes and/or percentage differences were calculated for key outcomes, on transformed data where applicable, and interpreted as follows: ≥ 0.2 = “small,” ≥ 0.5 = “medium,” ≥ 0.8 = “large.” Data are presented as mean ± SD for normally distributed data and geometric mean ± SD factor for log-normally distributed data. Normally distributed variables are displayed using linear axes and log-normally distributed variables on log-axes.

## Results

### Study 1: Relationship Between Tear AMPs and URTI Susceptibility

Eleven of 33 participants reported an episode of URI, of which nine were associated with a respiratory pathogen. Of the 22 participants who did not report URI, five were carrying respiratory pathogens at week 3. Therefore, nine participants with “URTI” (four contact lens wearers and five non-contact lens wearers), two “URS” participants with symptoms but negative virology (non-contact lens wearers), 17 “healthy” participants (three contact lens wearers and 14 non-contact lens wearers), and five “asymptomatic carriers” (positive virology but no symptoms, no contact lens wearers) were included in the analysis. Within the largest, healthy group, seven participants provided morning samples (10:00–12:00) and 10 afternoon (14:00–16:30) samples. Sub-analysis of the healthy group at two time points (pre- and post-monitoring) revealed no significant diurnal variation in AMP concentrations or secretion rates between participants sampled in the morning vs. afternoon (all *P* > 0.05, Table 1). Each of the other groups included samples taken in both morning and afternoon (URTI: three morning, six afternoon; URS: one morning, one afternoon; asymptomatic carriers: three morning, two afternoon.)

**Table 1:** Tear lysozyme (Lys) and lactoferrin (Lf) in Healthy participants who provided samples in the morning (7 participants) vs. afternoon (10 participants) at two time points.

**Variable**	**Morning (*n* = 14)**	**Afternoon (*n* = 20)**	***t (df)***	***P***	***d***
Lys-C mg·mL^−1^	0.56 ± 2.05	0.74 ± 1.97	1.19 (31)	0.24	0.43
Lys-SR μg·min^−1^	3.04 ± 3.10	2.10 ± 2.83	1.04 (31)	0.30	0.35
Lf-C mg·mL^−1^	2.90 ± 1.89	3.15 ± 2.26	0.61 (32)	0.54	0.09
Lf-SR μg·min^−1^	15.77 ± 2.84	8.56 ± 3.53	1.53 (32)	0.14	0.52

*One sample in the morning group was out of range of the assay for Lys-C and not included in the analysis of Lys. Data are geometric mean ± geometric SD factor. C, concentration; SR, secretion rate; t, t-statistic; df, degrees of freedom; d, Cohen's d effect size*.

Tear Lys concentration (C) and secretion rate (SR) were lower in participants with URTI (in samples collected during URTI) compared to healthy participants, with large Cohen's *d* effect sizes (Table 2). However, there were no significant differences in tear Lf between participants with URTI and healthy participants (Table 2). When all symptomatic (URTI + URI) or asymptomatic participants (healthy + asymptomatic carriers) were included in the analysis, the effect sizes between the groups were smaller but still “medium” (Table 2).

**Table 2:** Comparison of tear lysozyme (Lys) and lactoferrin (Lf) during pathogen-confirmed URTI (*n* = 9, samples collected during URTI) vs. participants who remained healthy throughout the monitoring period (*n* = 17, post-monitoring samples, pathogen-free at time of sampling), as well as all participants with symptoms (URTI + URS, *n* = 11) vs. all those without symptoms (healthy + asymptomatic carriers, *n* = 22).

**Variable**	**URTI**	**Healthy**	***t (df)***	***P***	***d***
Lys-C^a^ mg·mL^−1^	0.39 ± 0.17	0.96 ± 0.63	3.43 (18.5)	**0.003**	1.11
Lys-SR^b^ μg·min^−1^	0.83 ± 3.21	2.49 ± 2.92	2.30 (23)	**0.03**	0.96
Lf-C^b^ mg·mL^−1^	2.77 ± 1.26	2.96 ± 2.68	0.96 (20.8)	0.35	0.30
Lf-SR^b^ μg·min^−1^	6.54 ± 3.41	9.61 ± 3.88	0.90 (24)	0.38	0.37
**Variable**	**URTI + URS**	**Healthy + asymptomatic carriers**	***t (df)***	***P***	***d***
Lys-C^b^ mg·mL^−1^	0.36 ± 1.62	0.58 ± 2.44	2.69 (27.2)	**0.012**	0.77
Lys-SR^b^ μg·min^−1^	0.90 ± 3.02	1.83 ± 3.16	2.30 (30)	0.10	0.70
Lf-C^b^ mg·mL^−1^	2.74 ± 1.23	2.72 ± 2.41	0.57 (27.4)	0.57	0.21
Lf-SR^b^ μg·min^−1^	6.07 ± 3.72	8.27 ± 3.48	0.72 (31)	0.48	0.30

*One sample in the healthy group was out of range of the assay for Lys-C and not included in the analysis of Lys. C, concentration; SR, secretion rate; t, t-statistic; df, degrees of freedom; d, Cohen's d effect size*.

a *mean ± SD*;

b *geometric mean ± geometric SD factor. Bold text indicates p < 0.05*.

**Figure 1:**
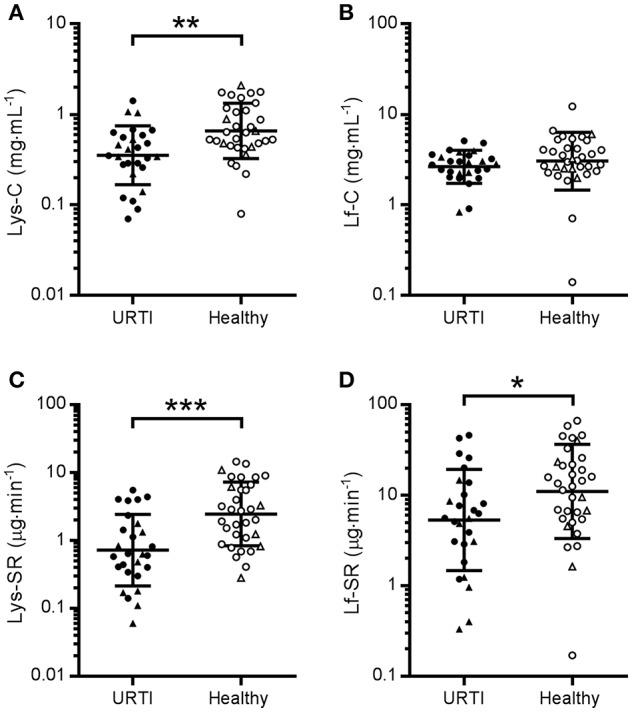
Tear lysozyme (Lys) and lactoferrin (Lf) in participants with pathogen-confirmed upper respiratory tract infection compared to healthy, pathogen-free controls. Pooled data from three time points in the URTI group (1 week before, during and after recovery from URTI, *n* = 9 × 3, closed symbols) and two time points in the Healthy group (beginning and end of 3-week monitoring period, *n* = 17 × 2, open symbols). •, non-contact lens wearers;▴, contact lens wearers; C, concentrations; SR, secretion rates. **(A)** Lys-C; **(B)** Lf-C; **(C)** Lys-SR and **(D)** Lf-SR. Data are geometric mean and SD factor. Difference between groups: ^*^P < 0.05, ^**^P < 0.01, ^***^P < 0.001.

Comparison of the grand mean of tear AMP concentrations and secretion rates in healthy participants (*n* = 17) at two time-points (pre- and post-monitoring), vs. participants with URTI (*n* = 9) at three time points (pre-URTI, during URTI and recovery) revealed that tear Lys-C was lower in the URTI group throughout the monitoring period (Figure 1A; *P* = 0.002, *d* = 0.85). Similarly, tear Lys-SR was lower in participants who reported URTI throughout the study (Figure 1C; *P* < 0.001, *d* = 0.99). A smaller difference in the grand mean of tear Lf-SR between URTI and healthy participants was also found (Figure 1D; *P* = 0.019, *d* = 0.62). There was no significant difference in Lf-C between groups (Figure 1B, *P* = 0.132, *d* = 0.38).

**Figure 2:**
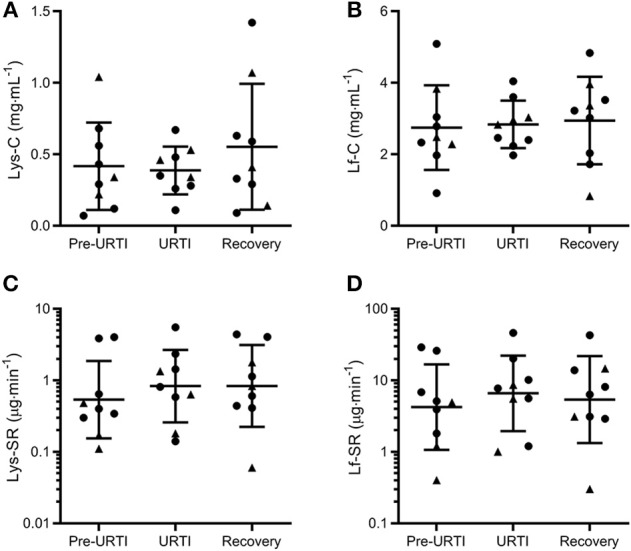
Tear lysozyme (Lys) and lactoferrin (Lf) 1 week before (Pre-URTI), during (URTI) and 4 weeks after (Recovery) an episode of upper respiratory tract infection. No differences in tear Lys or Lf were observed before or during URTI compared to recovery. Concentration data are mean and SD, secretion rates are geometric mean and SD factor. •, non-contact lens wearers;▴, contact lens wearers; C, concentrations; SR, secretion rates. **(A)** Lys-C; **(B)** Lf-C; **(C)** Lys-SR and **(D)** Lf-SR.

Repeated-measures analysis of the nine participants who reported pathogen-confirmed URTI revealed no differences in tear Lys or Lf either before or during URTI compared to when the same participants had been symptom-free for 4 weeks (Figure 2). These results remained if the two participants who reported symptoms but did not have positive viral cultures were also included (data not shown).

**Figure 3:**
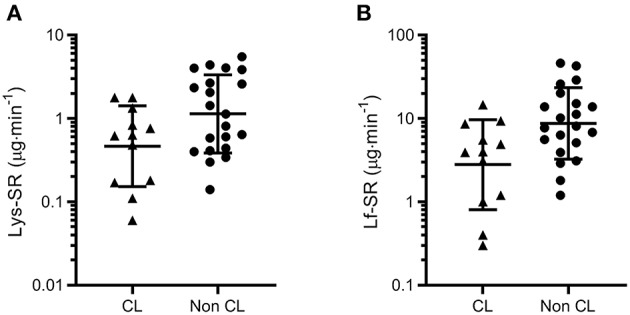
Tear lysozyme (Lys, **A**) and lactoferrin (Lf, **B**) secretion rates (SR) measured across three time points in symptomatic participants (URTI + URS, *n* = 11). Both tear Lys-SR and Lf-SR were significantly lower in contact lens wearers (*P* = 0.018 and *P* = 0.008, respectively). Data are geometric mean and SD factor.

Across the three time points combined in the symptomatic group (URTI + URS), tear Lys-SR and Lf-SR were also lower in contact lens wearers than non-contact lens wearers (12 vs. 21 time-points from 4 vs. 7 participants; *P* = 0.018 and *P* = 0.008 respectively, Figure 3).

Subsequently, to examine whether the results displayed in Figure 1 were an artifact of the differing proportions of contact lens wearers in each group, the analyses were repeated with contact lens wearers excluded. When contact lens wearers were excluded from the analysis, significant differences remained between the URTI and Healthy groups for Lys-C (*P* = 0.005) and Lys-SR (*P* = 0.012) but not for Lf-SR (*P* = 0.223) (data not shown).

### Study 2: Influence of Prolonged Exercise on Tear AMPs

**Figure 4:**
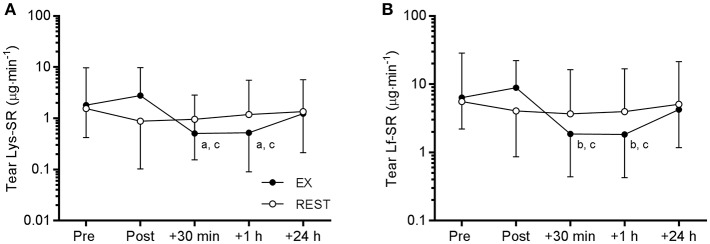
Tear lysozyme (Lys, **A**) and lactoferrin (Lf, **B**) secretion rate (SR) responses to 120 min exercise at ~65% VO_2peak_ (EX, closed circles) compared to seated rest control (REST, open circles). Significant difference between time points on EX: a, vs. Pre, *P* < 0.05; b, vs. Pre, *P* < 0.01; c, vs. Post, *P* < 0.001. Data are geometric mean and SD factor.

Tear Lf and Lys concentration were not influenced by exercise (data not shown). However, there was a time × trial interaction for Lf-SR [*F*_(4, 48)_ = 3.594, *P* = 0.012] and Lys-SR [*F*_(4, 48)_ = 3.521, *P* = 0.013). *Post-hoc* analyses revealed that Lf and Lys secretion rates were significantly reduced at 30 min and 1 h post-EX compared to pre- and immediately post-EX (Figure 4). Compared to pre-EX values, Lf-SR was 42% lower at 30 min post-EX (*d* = 0.91) and 1 h post-EX (*d* = 0.93). Similarly, Lys-SR was reduced by 49% at 30 min post-EX (*d* = 0.80) and by 48% at 1 h post-EX (*d* = 0.81) vs. pre-EX. There were no differences in tear Lf and Lys-SR between any of the time points on the REST trial.

### Study 3: Influence of Dehydration on Tear AMPs

**Figure 5:**
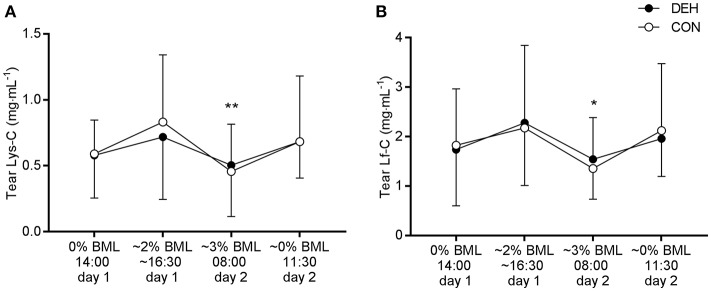
Influence of exercise-induced dehydration, followed by overnight fluid restriction (DEH) compared to euhydrated control (EUH) on tear lysozyme concentration (Lys-C, **A**) and lactoferrin concentration (Lf-C, **B**). Data are mean and SD. Main effect of time: ^*^P < 0.05, ^**^P < 0.01.

There was no influence of dehydration on tear AMP concentrations or secretion rates. However, we observed a main effect of time for Lys-C [*F*_(3, 36)_ = 5.108, *P* = 0.0048] and Lf-C [*F*_(3, 36)_ = 3.539, *P* = 0.024]. *Post-hoc* comparisons revealed that Lys-C and Lf-C were lower at 08:00 (~3% BML) than at 16:30 on day 1 (~2% BML; *P* < 0.05; Figure 5). There were no main effects of time for tear AMP secretion rates (data not shown).

## Discussion

This aim of this series of studies was to evaluate tear AMPs as clinically relevant biomarkers of immune function. Our primary aim in study 1 was to explore the clinical relevance of tear AMPs as markers of host defense, by investigating whether AMP secretion rates were associated with subsequent presentation of upper respiratory symptoms. We found that the participants who reported URTI during the monitoring period had lower tear AMP concentrations and secretion rates across all time points combined compared to those who remained healthy. These findings suggest that tear AMPs may be clinically relevant biomarkers of host defense. However, no change in tear AMP concentrations or secretion rates occurred before or during illness compared to when the same participants were illness-free, suggesting these markers have less potential to monitor week-to-week changes in immunity but may potentially be useful to determine immune status over a longer time-period. We previously demonstrated that tear secretory IgA has utility to detect changes in immune status in the days before presentation of URI (34). Thus, we suggested acute perturbations in tear IgA may have utility to predict susceptibility to URTI within the forthcoming days. On the other hand, the findings from this study suggest that tear Lf and Lys may be better suited to identify individuals who are illness-prone or chronically susceptible to URTI. Future studies may therefore wish to investigate whether tear Lys and Lf are chronically lower in participants prone to recurrent URTI over a longer time-period.

Prolonged exercise typically causes brief post-exercise immunomodulatory effects (41). Whether or not such responses may be detrimental to immune competence, via the so-called “open window” hypothesis, has been recently reviewed elsewhere (16). Focusing specifically on mucosal AMPs, previous studies have demonstrated no change (37, 42, 43), an increase (44, 45) or a decrease (46) in salivary AMP secretion rates after prolonged exercise. Decreases in salivary AMP concentration that have been observed post-exercise may be offset by increases in flow rate (42, 47). In study 2, we saw no change in tear AMP concentration or secretion rate immediately post-exercise, but observed large reductions in tear Lf and Lys secretion rates between 30 min and 1 h post-exercise. Since tear AMP concentrations were not affected by prolonged exercise, this response likely occurred as a result of downregulation of lacrimal gland secretory activity, that is, a reduction in the output of proteins, water and electrolytes (27). A single bout of prolonged exercise is known to exhibit a biphasic immune response characterized by an acute increase in total lymphocyte count followed by a decrease to below pre-exercise levels in the post-exercise recovery period (48, 49). Elevated cortisol post-exercise has been shown to maintain an increase in neutrophil counts for up to 2 h post-exercise, accompanied by an increased generation of reactive oxygen species (50). Prolonged exercise may also increase a range of circulating cytokines and chemokines including IL-6, IL-8, IL-10, and TNF-*A* for up to 3 h post-exercise (48, 51, 52). Although we did not measure circulating hormones, leukocytes or cytokines in the present study, *in-vitro* studies have demonstrated that oxidative stress, steroid hormones and pro-inflammatory cytokines can inhibit lacrimation (31, 32). Since lacrimal gland activity is primarily regulated by the autonomic nervous system, alterations to autonomic balance during the post-exercise recovery period may also have directly influenced tear output. Thus, while the mechanism by which tear AMP availability was reduced post-exercise is not clear from this study, our findings illustrate a transient decrease in lacrimal gland secretion and thus availability of AMPs at the ocular surface during the post-exercise recovery period that led to a localized, albeit temporary reduction in immune competence.

Since a majority of studies report no influence of mild dehydration on cellular immune parameters (53), mild dehydration (1–3% BML) has often been viewed as confounding variable (as opposed to a stressor) when considering mucosal responses to exercise. Whilst one study reported temporary reductions in salivary AMP secretion rates following mild exercise-induced dehydration (47), another study reported no influence of fluid restriction-induced dehydration on AMP secretion rates post-exercise (38). As expected in study 3, we found no evidence to suggest dehydration influences tear AMP concentration or secretion rate, and can thus conclude that tear AMPs are robust to alterations in hydration status.

In study 1 we did not find any evidence of diurnal variation between samples collected mid-morning and mid-afternoon in healthy participants, but in study 3 we observed that tear AMP concentrations were lower first-thing in the morning (08:00) compared to late afternoon (16:30) in the same participants. This diurnal variation was not seen in tear AMP secretion rates. Whilst we did not specifically design either study to investigate diurnal variation, previous studies have reported that tear Lys has a tendency to be lower in the morning and higher in the evening, as seen in study 3 (54). This could perhaps be a consequence of lower lacrimal gland secretion at night, leading to a lower proportion of tear Lys and Lf in the closed-eye state (55) which may take some time to resolve after waking.

In study 1 we also saw that tear AMP secretion rates were lower in contact lens wearers, and that there was a higher proportion of contact lens wearers in the URTI group (4/9) than the pathogen-free, healthy group (3/17). This is consistent with previous studies that have shown increased tear osmolarity and lower tear volume in contact lens wearers (56). Although contact lens wear is an established risk factor for microbial keratitis (57), to our knowledge no studies have investigated whether contact lens wear increases URTI incidence, which is feasible since the eye is a portal for inoculation of viral pathogens such as influenza (4). It would therefore be interesting to further explore whether the use of contact lenses is in itself a risk factor for URTI, for example by reducing the secretion rates of tear AMPs or by facilitating transmission of pathogens via the ocular surface.

### Strengths and Limitations

A strength of this series of studies is the thorough experimental approach employing both well-controlled repeated-measures crossover designs to explore the effect of dehydration and exercise on tear AMPs as well as the prospective cohort design of study 1. In study 1, given the geographic proximity of the participants to each other, we can assume that that most, if not all, participants will have been exposed to URTI during the monitoring period. We also had a good representation of URTI in the sample with almost one-third of participants self-reporting URI within the monitoring period, of whom nine of eleven returned positive virology tests. This is a higher rate of positive tests than in previous studies in athletes that have typically identified pathogens in only 30–40% of self-reported URI episodes (40, 58). As only a limited panel of bacteria and viruses was used, it is possible that the two participants who reported symptoms but with a negative virology test could also have contracted an infection that was not identified by the diagnostic test, or that the symptoms arose from a non-infectious origin. Thus, we argue that studying both the populations who reported episodes of URI as a whole (symptomatic group) as well as the sub-population who reported symptoms and positive virology (URTI group) is pertinent to understanding the utility of Lf and Lys as biomarkers of illness risk. Nevertheless, with our cohort of 40 participants, the statistical power of the resulting URTI and Healthy groups was sufficient only to detect large effects. Lifestyle variables such as alcohol and tobacco intake, exercise and sleep were not controlled in study 1, so we consider it a strength for the clinical relevance of tear AMPs that we saw differences between the URTI and Healthy groups in spite of the background “noise” of the variables that were not controlled.

A potential limitation of the sampling technique and/or in the use of tears to assess AMPs was the large variability in tear flow rates. Although previous studies suggest that concentrations of tear Lys and Lf are similar in unstimulated and reflex tears (55, 59), there was substantial variability in tear flow rates both between- and within-participants in all studies. Samples obtained with high flow rates may therefore have led to high outliers in AMP secretion rates. Additionally, there are some challenges in measuring the flow rate of tears, since some of the initial sample may be obtained from basal tears already residing on the lower lid of the eye, a volume estimated at around 7 μL (26). However, it is impractical to drain the eye of tears before beginning collection, as may be achieved to some extent in saliva sampling by swallowing before commencing sample collection. Conversely, we had difficulty obtaining sufficient volumes of tear sample from a few participants, leading to longer collection times. Whether these participants were not producing tears or that the tears were difficult to access remains unclear. This may also apply to contact lens wearers, in whom AMP secretion rates were lower within the participants who reported URS in study 3. Several of these constraints could be overcome by using AMP concentrations as primary outcome variables, as they are less affected by tear flow rates. On the other hand, it is highly possible the *availability* of tear AMPs at the ocular surface is more important for host defense, indicated by secretion rates. Indeed, in the present study, only AMP secretion rates were significantly lower following prolonged exercise; a response that we may expect to observe in a clinically relevant immune biomarker. There is also potential for nanotechnology to facilitate the development of continuous monitoring devices, such as “smart” contact lenses (33, 60) or point-of-care devices that could improve the ability to measure tear flow as well as tear fluid composition.

## Conclusions

This series of studies set out to evaluate the potential clinical relevance and utility of the tear fluid AMPs Lf and Lys as minimally-invasive biomarkers of mucosal immune competence. We observed that tear Lys concentration and Lys and Lf secretion rates were lower in participants who reported an episode of URTI within a 3-week monitoring period, compared to individuals who remained healthy. Tear AMP secretion rates were also temporarily reduced 30 min to 1 h after prolonged treadmill exercise and were robust to any confounding effect of dehydration on flow rates at mucosal surfaces. We also found that tear AMP secretion rates were lower in contact lens wearers, albeit among a small cohort. Thus, our observations serve to highlight avenues for further study but follow-up studies will be important to verify these preliminary findings. Collectively, this series of studies provides an initial demonstration that tear AMPs, especially secretion rates, may be clinically relevant markers of mucosal immune competence. It is possible that progress in nanotechnology and microfluidics will facilitate development of devices to improve measurement of tear flow and composition in the future.

## Data Availability

The datasets generated for this study are available on request to the corresponding author.

## Ethics Statement

All studies received approval from the Bangor University School of Sport, Health and Exercise Sciences Ethics Committee (application numbers S/PhD08-14/15, S/PhD11-12/13 and S/PhD09-13/14). Participants provided written, informed consent before taking part, and all test procedures were conducted in accordance with the Declaration of Helsinki.

## Author Contributions

HH and JE collected and analyzed the data and HH drafted the manuscript. HH, JE and NW contributed to conception and design of each of the studies, critically reviewed the manuscript, approved the final version of the work and agree to be accountable for all aspects of the work.

### Conflict of Interest Statement

For study 3, HH was the recipient of a Graduate Student Research Grant from the European Hydration Institute. The remaining authors declare that the research was conducted in the absence of any commercial or financial relationships that could be construed as a potential conflict of interest.
